# Development and validation of a nomogram for predicting the probability of arthroplasty in patients with osteonecrosis of the femoral head after fibular strut grafting

**DOI:** 10.1080/07853890.2026.2734061

**Published:** 2026-10-08

**Authors:** Haitao Zhang, Zequan Luo, Jiayi Zhu, Guowen Bai, Jiaming Zeng, Ke Jie, Qiang Wan, Xinyu Qi, Jie Li, Jianchun Zeng, Zhiyuan Wang, Kaifeng Cheng, Hang Quan, Yirong Zeng, Wenjun Feng, Yijin Li, Jinlun Chen

**Affiliations:** a Department of Orthopaedics, The First Affiliated Hospital of Guangzhou University of Chinese Medicine, Guangzhou, Guangdong, China; b Guangzhou University of Chinese Medicine, Guangzhou, Guangdong, China; c Department of Orthopaedics, Foshan Traditional Chinese Medicine Hospital, Foshan, China

**Keywords:** Fibular strut grafting, risk factors, nomogram, osteonecrosis of the femoral head, total hip arthroplasty

## Abstract

**Background:**

While fibular strut grafting (FSG) is effective for osteonecrosis of the femoral head (ONFH), some patients still progress to total hip arthroplasty (THA). This study aimed to identify risk factors for FSG failure and develop a validated nomogram to predict the mid-long term survival rate after FSG.

**Methods:**

Data on patients with ONFH who underwent FSG were collected in our institution from February 2009 to December 2018. Independent predictors were identified using univariable and multivariable Cox regression analyses. Kaplan-Meier analysis was used to estimate survival after FSG. A nomogram predicting  failure, defined as conversion to THA, was developed from the selected predictors and internally validated using the concordance index (C index), the Receiver Operating characteristic (ROC) curves, and calibration curves.

**Results:**

Among 416 eligible patients, 55 converted to THA. The 5-, 8-, and 10-year survival rates after FSG were 89.7%, 83.6%, and 82%, respectively. Age, smoking, Fibrinogen (FIB), hypertension, diabetes, and ARCO stage were independent predictors of failure. Subsequently, a nomogram model was developed incorporating 7 predictors (including JIC classification). Its C-index was 0.734 (95%CI 0.66–0.80). The area under the curves (AUCs) of the nomogram predicted for 5-, 8-, and 10-year were 0.727, 0.735, and 0.735, respectively. Predicted and observed survival rates agreed closely.

**Conclusion:**

The internal validation shows that the nomogram model has acceptable discriminative ability and good accuracy. It may support clinical application, effectively optimize perioperative management, and help doctors and patients to avoid FSG failure.

## Introduction

Osteonecrosis of the femoral head (ONFH) is a crippled disease orthopedic disease caused by a variety of factors [1,2]. Without intervention, the blood supply to the femoral head is severely disrupted, and a large number of the active components of the bone marrow die off, followed by femoral head collapse and even progression to severe hip pain and dysfunction [1–4]. Substantially, most patients inevitably face the fate of total hip arthroplasty (THA). However, orthopedic surgeons are more inclined to select hip-preserving surgery to delay performing THA due to various complications in young patients [5]. Currently, femoral osteotomy, core decompression, vascularized bone grafting, non-vascularized bone grafting, stem cells, and other hip-preserving surgeries have been proposed and widely applied in clinics to improve the joint function of young patients [3,6].

The mechanism of fibular strut grafting (FSG) is predominantly to defer or prevent the collapse of the femoral head by removing necrotic bone, inducing microcirculation repair of the femoral head, and providing mechanical stability [7,8]. Our previous studies have confirmed that FSG is definitely effective in treating ONFH [9,10]. Due to its advantages of being minimally invasive (requiring no vascular anastomosis and typically utilizing allografts), technically straightforward, associated with fewer complications, and cost-effective, FSG has become an increasingly popular technique among all hip-preserving surgeries [11,12]. However, some patients still underwent THA after FSG, even when it is performed with advanced techniques [13]. Hence, it is crucial to predict the prognosis of patients with non-vascularized allogeneic fibular grafts and enhance the long-term survival rates. To date, a few scholars have reported the risk factors related to the failure of FSG in the treatment of ONFH, and there are also a few specialized studies on the establishment of personalized predictive models to accurately guide clinical application. In recent years, the nomogram predictive model has been widely used as a mature means to predict the prognosis of patients in the field of medicine [14–17]. In the nomogram predictive models, multiple risk factors can be scientifically and comprehensively combined to predict the incidence of THA after FSG, and the results can be visually presented to doctors and patients.

Although risk factors for hip-preserving surgery failure are well documented, a comprehensive predictive model specific to FSG failure remains lacking. Given the inherent limitations of the retrospective design, variable prioritization was performed based on established pathophysiological mechanisms [18,19], including microcirculatory disturbance, hypercoagulable state, and nicotine effect. These included comorbidities (hypertension, diabetes), smoking, hyperfibrinogenemia, and radiographic staging (ARCO, JIC).

Consequently, the purposes of this retrospective study were to determine the risk factors of failure (THA) in patients with ONFH after FSG and to develop and validate an internally validated nomogram model to systematically evaluate the mid-long term survival rate of patients with ONFH after FSG.

## Materials and methods

### Inclusion and exclusion criteria

Data on patients with ONFH who underwent FSG in our institution from February 2009 to December 2018 were retrospectively collected and analyzed on March 2nd, 2025. The reporting of this observational study was conducted in accordance with the STROBE (Strengthening the Reporting of Observational Studies in Epidemiology) statement [20], and the corresponding STROBE checklist is provided in supplementary Table 2. The study was approved by the Ethics Committee of The First Affiliated Hospital of Guangzhou University of Chinese Medicine (Approval No. K-2022-003) and was conducted in strict accordance with the principles of the Declaration of Helsinki. All participants have obtained written informed consent.

Inclusion criteria include: (1) diagnosed with ONFH; (2) underwent FSG; (3) follow-up for more than two and a half years. Exclusion criteria are as follows: (1) ONFH caused by pathological femoral neck fracture; (2) loss of follow-up patients; (3) lack of data; (4) death not related to FSG; (5) patients under 18 years of age were excluded to ensure a homogeneous adult cohort. This is because skeletal immaturity, distinct etiology, and potential differences in postoperative rehabilitation compliance in adolescents could introduce confounding variables affecting the model’s validity for the adult ONFH population.

### Surgical procedures and the postoperative rehabilitation program

After the anesthesia is satisfactory, the patient lies on his back on the operating table. The hip pad on the operative side is 2-5cm high to facilitate the operation. A 6-10cm incision was made downward at the apex of the lateral greater trochanter of the thigh. Under C-arm fluoroscopy, a 2.5 mm drill was drilled from 2 cm below the greater trochanter to 0.4–0.5 cm under the anterolateral cartilage of the femoral head. Along the guide needle, a reaming reamer from small to large sizes was used to decompress the hole to the subcortical 0.5 cm. The allogeneic cancellous bone particles were packed into the necrotic area of the femoral head by a decompression tunnel. Subsequently, the allogeneic fibular strips were implanted into the decompression tunnel to the top to raise the necrotic area of the femoral head. Although the use of AO screws for internal fixation was left to the discretion of the chief surgeon, the core surgical procedure—including the entry point, drilling trajectory, decompression extent, and depth of fibular strut implantation—was strictly standardized across all operators. This standardization was maintained through regular multidisciplinary training sessions and intraoperative C-arm fluoroscopy guidance to ensure consistency in biomechanical support, regardless of individual surgeon preferences regarding screw fixation.

The drainage tube was removed on the second day after the operation, and the quadriceps muscle contraction and hip flexion and extension began after pain relief. Patients were required to use double crutches for weight-free exercise within 6 weeks after the operation. Partial weight bearing can be carried out after 3 months. After 6 months, whether weight bearing is allowed or not depends on the degree of the necrotic area of the femoral head (but heavy manual labor is prohibited). Maintain skin traction of the affected limb 12 h a day for at least 6 months.

### Data collection

The study data included baseline data, operation-related variables, serum biochemical marker levels, and radiological parameters. Baseline data, operation-related variables, and serum biochemical marker levels were collected from the electronic medical records database. Baseline data included sex, age, body mass index (BMI), side, the American Association of Anesthesiologists (ASA) classification, preoperative necrotic factors, smoking, hypertension, and diabetes. Operation-related variables included screw fixation, cooperation with platelet-rich plasma (PRP), and operation time. Serum biochemical markers levels included white blood Cell Count (WBC), total lymphocyte count (LYM), hemoglobin (HGB), platelet (PLT), fibrinogen (FIB), and albumin (ALB).

Regarding radiological parameter measurement, it was speculated that the position of the allogeneic fibular strip determines the biomechanical stability of the femoral head, which may be related to the prognosis of postoperative patients. Therefore, on the postoperative anteroposterior radiograph, the height between the top of the allograft fibula strip and the subchondral bone surface of the femoral head (AH), and the distances between the bone cortex on both sides of the fibula strip and the femoral head and neck junction (AD1 and AD2) were respectively measured. The ratio of AD1 to AD2 was defined as ADR to determine the position of the allogeneic fibular strip on the anteroposterior radiographs. On the frog-leg lateral radiographs, the height between the top of the allograft fibula strip and the subchondral bone surface of the femoral head (FH), and the distances between the bone cortex on both sides of the fibula strip and the femoral head and neck junction (FD1 and FD2) were respectively measured. The ratio of FD1 to FD2 was defined as FDR to determine the position of the allogeneic fibular strip on the frog-leg lateral radiographs (Figure 1). The 2019 revised Association Research Circulation Osseous (ARCO) stage [21] and Japanese Investigation Committee (JIC) classification [22] were assessed independently by two senior orthopedic surgeons blinded to clinical outcomes, with any discrepancies resolved by a third adjudicator. All radiological assessments were performed according to preoperative and postoperative radiography. Patients were followed up by telephone communication, outpatient visits, and WeChat. The endpoint of the study was THA.

**Figure 1.**
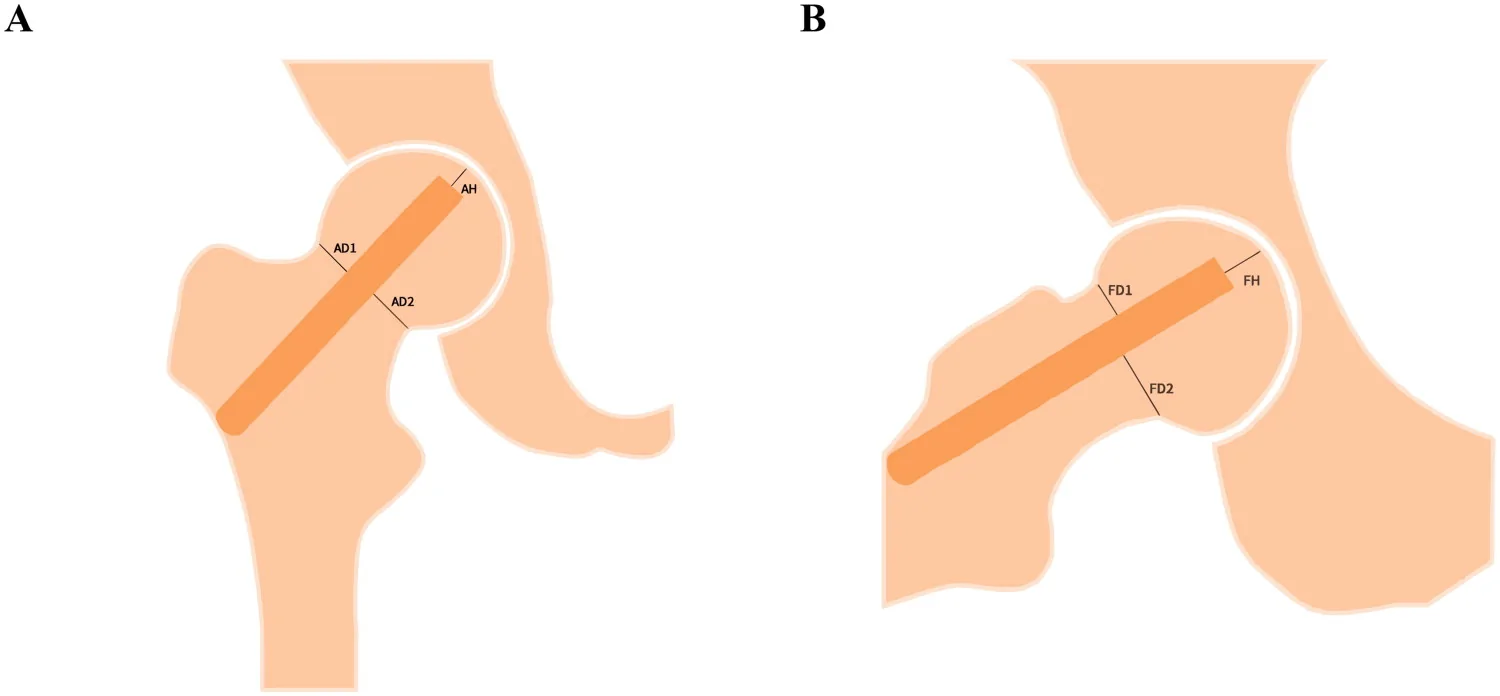
Diagram of fibula allograft propping. On the postoperative anteroposterior, we respectively measured the height between the top of allograft fibula strip and the subchondral bone surface of the femoral head (AH), the distance between the bone cortex on both sides of the fibula strip and the femoral head and neck junction (AD1 and AD2). The ratio of AD1 to AD2 was defined as ADR to determine the position of the allogeneic fibular strip on the anteroposterior radiographs. On the frog-leg lateral radiographs, we respectively measured the height between the top of allograft fibula strip and the subchondral bone surface of the femoral head (FH), the distance between the bone cortex on both sides of the fibula strip and the femoral head and neck junction (FD1 and FD2). The ratio of FD1 to FD2 was defined as FDR to determine the position of the allogeneic fibular strip on the frog-leg lateral radiographs.

### Statistical analysis

All data analysis and images production were performed by IBM SPSS Statistics version 23 (IBM, Armonk, New York), R software (version 4.1.1, R Foundation for Statistical Computing, Austria), and GraphPad Prism version 8. Continuous variables are presented as mean ± standard deviation. Categorical variables are described as frequency (percentages). Continuous variables are presented as mean ± standard deviation (SD) or median (interquartile range [IQR]) based on their distribution. Normality of continuous variables was assessed using the Anderson-Darling test. Only age followed a normal distribution (*p* > 0.05); therefore, group comparisons were performed using the independent samples t-test for age and the Mann-Whitney U test for all other non-normally distributed continuous variables. For continuous variables, missing values were imputed using mean imputation. For categorical variables, missing values were imputed using mode imputation.

The risk factor screening was divided into two steps. Firstly, candidate variables were screened using univariate Cox regression analysis with a relaxed inclusion threshold of *p* < 0.1. This approach is widely recommended in clinical prediction model building to avoid omitting potentially important confounding variables that may not reach statistical significance in univariate analysis but become significant contributors in a multivariable context (e.g. through confounding effects or interactions) [23]. Unless otherwise specified, categorization of continuous variables for modeling followed the left-closed, right-open principle (e.g. [a, b)). Subsequently, these significant variables were selected to construct a multivariable Cox proportional hazard regression model. The final risk factors for THA after FSG are determined according to *p* < 0.05 in multivariate COX regression analysis. A nomogram model that could provide survival rates at multiple time points was established using significant predictors screened by multivariate Cox regression analysis. The classification variables were compared using the chi-square test or Fisher’s exact test. The Kaplan-Meier survival curve was plotted on the basis of predictors in the nomogram model and follow-up outcomes. The concordance index (C index) and the Receiver Operating characteristic (ROC) curves at 5, 8, and 10 years were used to evaluate the discrimination ability of the nomogram model. Based on the internal verification of 1000 bootstrap resampling, the calibration plots of 5, 8, and 10 years were used to assess the calibration effect of the nomogram model.

## Results

### Patient baseline characteristics

The initial 477 patients were identified. After excluding 5 cases of death, 48 cases of loss of follow-up, 6 cases of missing data (missing data on whether THA occurred), and 2 cases of ONFH due to pathological femoral neck fracture, the remaining 416 patients met the criteria and were included in the study for analysis. The patient selection process is summarized in Supplementary Figure 2 and the comparison of patient data was summarized in Table 1. There were 324 male patients (77.9%) and 92 female patients (22.1%). The mean age of patients was 35.2 ± 9.00 (range 20 to 61). The mean follow-up time was 76.8 ± 37.2 months. At the last follow-up, fifty-five patients underwent THA and were deemed to have failed. There was no significant difference in age (continuous variable) between the THA and non-THA groups. However, when age was grouped into categorical variables, there was a significant difference between the two groups. HGB, FIB, ALB, ARCO, JIC, and Hypertension were also significantly different between the two groups.

**Table 1.** Demographics and clinical characteristics of patient. Categorical variables were compared using the Chi-square test or Fisher’s exact test (F), where (F) denotes Fisher’s exact test. Continuous variables were compared using the Mann-Whitney U test. Unless otherwise specified, categorization of continuous variables follows left-closed, right-open intervals (e.g., [a, b]).

Variable	Total (*n* = 416)	Non-Arthroplasty (*n* = 361)	Arthroplasty (*n* = 55)	*p* value
Age (y)	35.27 ± 9.00	34.98 ± 9.19	37.20 ± 7.43	0.051
Age (y)				**0.041 (F)**
18-40	276 (66.3)	247 (68.4)	29 (52.7)	
40–55	134 (32.2)	108 (29.9)	26 (47.3)	
≥55	6 (1.4)	6 (1.7)	0 (0)	
Gender, n (%)				0.181
Male	324 (77.9)	285 (78.9)	39 (70.9)	
Female	92 (22.1)	76 (21.1)	16 (29.1)	
BMI (kg/m^2^)				0.505 (F)
<18.5	22 (5.3)	19 (5.3)	3 (5.5)	
18.5-24	229 (55.0)	195 (54)	34 (61.8)	
24-28	129 (31.0)	113 (31.3)	16 (29.1)	
≥28	36 (8.7)	34 (9.4)	2 (3.6)	
Side, n (%)				0.330
Left	209 (50.2)	178 (49.3)	31 (56.4)	
Right	207 (49.8)	183 (50.7)	24 (43.4)	
Operation time (min)	107.17 ± 40.78	106.57 ± 40.10	111.16 ± 45.19	0.754
White blood cell (10^9/L)				1.000 (F)
<4	3 (0.7)	3 (0.8)	0 (0)	
4-10	372 (89.4)	321 (88.9)	51 (92.7)	
≥10	41 (9.9)	37 (10.2)	4 (7.3)	
Total Lymphocyte Count (10^9/L)				0.907 (F)
<1.6	84 (20.2)	74 (20.5)	10 (18.2)	
1.6-4	329 (79.1)	284 (78.7)	45 (81.8)	
≥4	3 (0.7)	3 (0.8)	0 (0)	
Hemoglobin (g/L)				**0.020 (F)**
<120	50 (12.0)	37 (10.2)	13 (23.6)	
120-160	338 (81.3)	300 (83.1)	38 (69.1)	
≥160	28 (6.7)	24 (6.6)	4 (7.3)	
Platelet (10^9/L)				0.619 (F)
<100	4 (1.0)	3 (0.8)	1 (1.8)	
100-300	346 (83.2)	300 (83.1)	46 (83.6)	
≥300	66 (15.9)	58 (16.1)	8 (14.5)	
Fibrinogen (g/L)				**0.028 (F)**
2-4	349 (83.9)	309 (85.6)	40 (72.7)	
≥4	67 (16.1)	52 (14.4)	15 (27.3)	
Albumin (g/L)				**0.017 (F)**
<40	43 (10.3)	32 (8.9)	11 (20.0)	
40–55	373 (89.7)	329 (91.1)	44 (80.0)	
AH (mm)				1.000 (F)
<10	159 (38.2)	137 (38)	22 (40.0)	
10-20	215 (51.7)	188 (52.1)	27 (49.1)	
≥20	42 (10.1)	36 (10.0)	6 (10.9)	
ADR				0.451 (F)
<1	378 (90.9)	326 (90.3)	52 (94.5)	
≥1	38 (9.1)	35 (9.7)	3 (5.5)	
FH (mm)				0.333 (F)
<10	187 (45)	165 (45.7)	22 (40.0)	
10-20	190 (45.7)	160 (44.3)	30 (54.5)	
≥20	39 (9.4)	36 (10.0)	3 (5.5)	
FDR				0.858 (F)
<1	330 (79.3)	287 (79.5)	43 (78.2)	
≥1	86 (20.7)	74 (20.5)	12 (21.8)	
ARCO, n (%)				
II	218 (52.4)	197 (54.6)	21 (38.2)	**<0.001**
IIIA	134 (32.21)	119 (33.0)	15 (27.3)	
IIIB	64 (15.4)	45 (12.5)	19 (34.5)	
JIC, n (%)				**0.016** (F)
A	15 (3.61)	14 (3.9)	1 (1.8)	
B	42 (10.1)	37 (10.2)	5 (9.1)	
C1	118 (28.4)	111 (30.7)	7 (12.7)	
C2	241 (57.9)	199 (55.1)	42 (76.4)	
Screw, n (%)				0.585
No	241 (57.9)	211 (58.4)	30 (54.5)	
Yes	175 (42.1)	150 (41.6)	25 (45.5)	
Platelet rich plasma, n (%)				0.644
No	383 (92.1)	331 (91.7)	52 (94.5)	
Yes	33 (7.9)	30 (8.3)	3 (5.5)	
Stem cell, n (%)				1.000 (F)
No	408 (98.1)	354 (98.1)	54 (98.2)	
Yes	8 (1.9)	7 (1.9)	1 (1.8)	
ASA, n (%)				0.871 (F)
I	191 (45.9)	167 (46.3)	24 (43.6)	
II	221 (53.1)	190 (52.6)	31 (56.4)	
III	4 (1.0)	4 (1.1)	0 (0)	
Factor, n (%)				0.633
Glucocorticoid	167 (40.1)	147 (40.7)	20 (36.4)	
Alcoholic	147 (35.3)	123 (34.1)	24 (43.6)	
Traumatic	39 (9.4)	35 (9.7)	4 (7.3)	
Idiopathic	63 (15.1)	56 (15.5)	7 (12.7)	
Smoking				0.053
No	309 (74.3)	274 (75.9)	35 (63.6)	
Yes	107 (25.7)	87 (24.1)	20 (36.4)	
Hypertension, n (%)				**<0.001**
No	405 (97.4)	356 (98.6)	49 (89.1)	
Yes	11 (2.6)	5 (1.4)	6 (10.9)	
Diabetes, n (%)				0.391 (F)
No	410 (98.6)	357 (98.9)	53 (96.4)	
Yes	6 (1.4)	4 (1.1)	2 (3.6)	

### Prognostic nomogram and survival

The proportional hazards assumption was satisfied for the final model (global Schoenfeld test *p* = 0.6; all individual *p* > 0.05; Supplementary Table 1). Based on this validated model, univariate Cox regression results (*p* < 0.1) showed that the candidate risk factors included age, ARCO, ALB, FIB, HGB, smoking, hypertension, and diabetes. Multivariate Cox regression analyses (*p* < 0.05) identified ultimately the following 6 independent predictors: Age (40–55 y, HR, 2.32; 95% CI, 1.32–4.08; *p* = 0.003), smoking (HR, 2.32; 95% CI, 1.26–4.24; *p* = 0.007), FIB (≥4, HR, 1.97; 95% CI, 1.05–3.68; *p* = 0.034), hypertension (HR, 4.43; 95% CI, 1.71–11.48; *p* = 0.002), diabetes (HR, 7.62; 95% CI, 1.71–34.05; *p* = 0.009), and ARCO stage (IIIB, HR, 2.72; 95% CI, 1.44–5.17; *p* = 0.002) (Table 2). To evaluate the contribution of JIC classification, we compared the full model (7 predictors) with a reduced model excluding JIC (6 predictors). The 5-year AUC was slightly higher in the full model (0.727 vs. 0.719), whereas the 8-year (0.735 vs. 0.761) and 10-year (0.735 vs. 0.795) AUCs were lower in the reduced model (Figure 5; Supplementary Figure 1). Although JIC did not reach statistical significance in the multivariate Cox regression (*p* > 0.05), we retained JIC in the final nomogram primarily based on strong clinical consensus regarding its role in assessing biomechanical stability. The nomogram model (consisting of 7 predictors) for predicting failure after FSG is shown in Figure 2. The 5-year, 8-year, and 10-year survival rates of patients after FSG were 89.7%, 83.6%, and 82%, respectively (Figure 3a). Kaplan-Meier survival curves grouped by independent predictors are demonstrated in Figure 3b-h.

**Figure 2.**
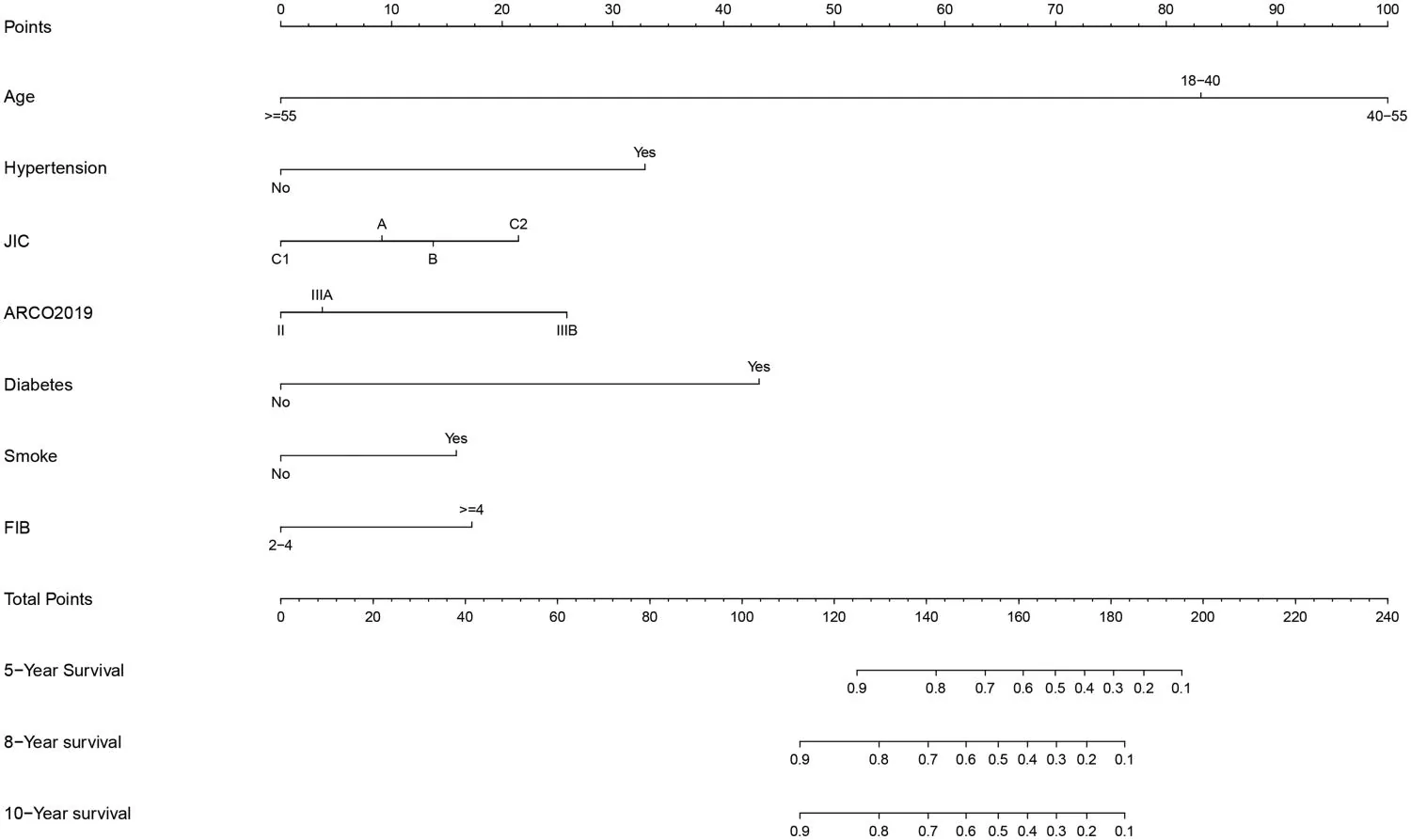
The nomograms model (consisting of 7 predictors) of predicting failure after fibula allograft propping. The corresponding score for each factor is based on the condition of the patient, which can be determined by making a vertical line upwards. Add all the scores to get the total score, then find the corresponding point on the total point axis, and make a vertical line down to predict the survival failure probabilities after fibula allograft propping. Unless otherwise specified, categorization of continuous variables follows left-closed, right-open intervals (e.g. [a, b]).

**Figure 3.**
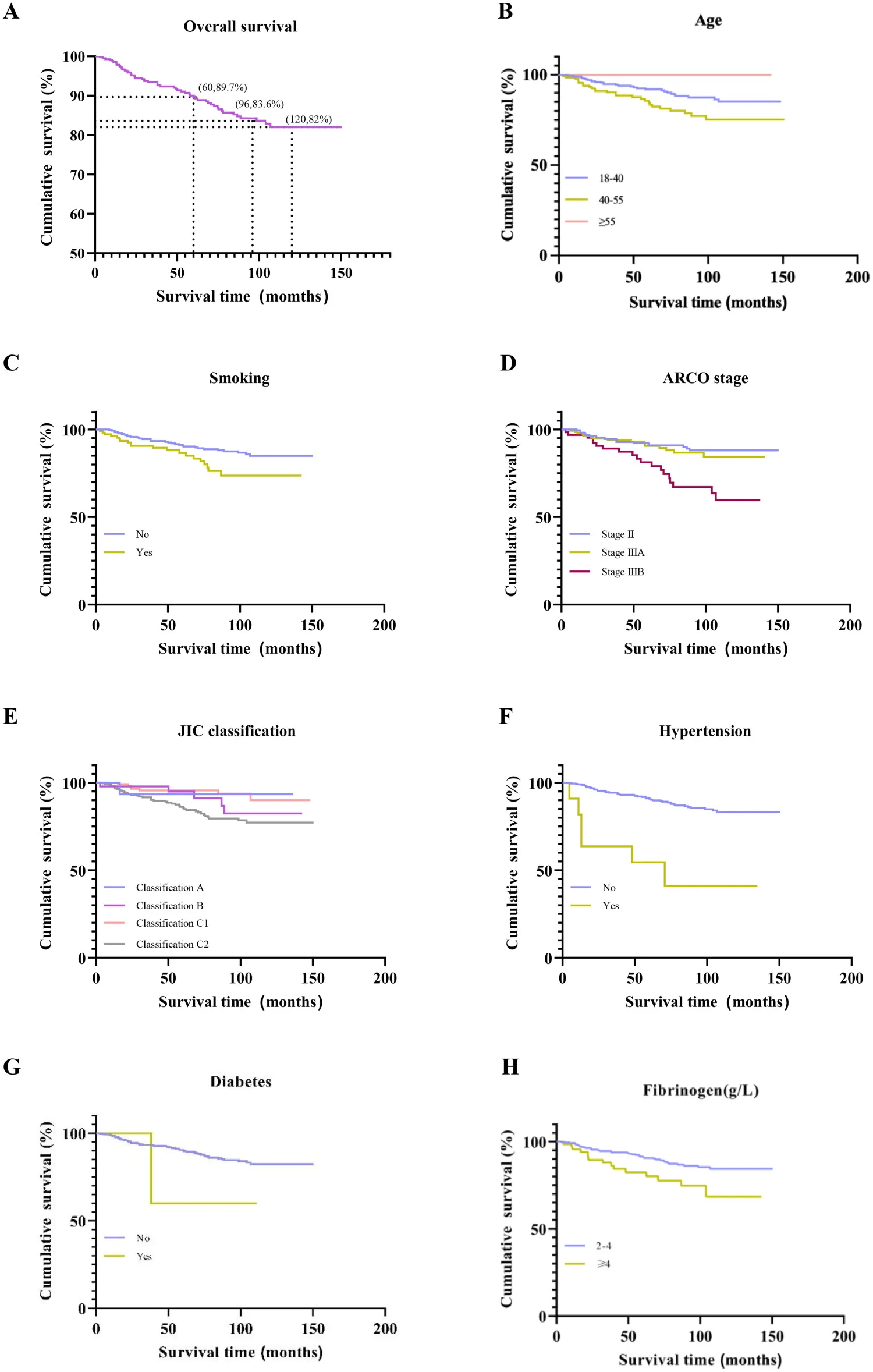
The Kaplan-Meier survival curves for the overall cohort (A) and significant independent predictors identified to be associated with THA after FAGP. Independent predictors included (B), Age (C), Smoking (D), ARCO stage (E), JIC classification (F), Hypertension (G), Diabetes (H), Fibrinogen.

**Table 2.** Univariate and multivariate analyses of risk factors. Categorical variables were compared using the Chi-square test or Fisher’s exact test (F), where (F) denotes Fisher’s exact test. Continuous variables were compared using the Mann-Whitney U test. Unless otherwise specified, categorization of continuous variables follows left-closed, right-open intervals (e.g., [a, b]).

	Univariate analysis		Multivariate analysis	
Variables	*p* value	HR (95% CI)	*p* value	HR (95% CI)
Gender				
female	–	Reference	–	–
male	0.270	1.39 (0.78–2.48)	–	–
Age				
** 18–40**	–	Reference	–	Reference
40–55	**0.017**	1.90 (1.12–3.23)	**0.003**	2.32 (1.32–4.08)
≥55	0.996	0.00 (0.00-Inf)	0.996	0.00 (0.00-Inf)
BMI				
<18.5	–	Reference	–	–
18.5-24	0.945	0.96 (0.29–3.13)	–	–
24-28	0.666	0.76 (0.22–2.62)	–	–
≥28	0.269	0.36 (0.06–2.18)	–	–
Side				
Left	–	Reference	–	–
Right	0.434	0.81 (0.47–1.4)	–	–
WBC				
<4	**-**	Reference	**-**	–
4-10	0.995	3405,637 (0.00-Inf)	**-**	–
≥10	0.995	2586,053 (0.00-Inf)	**-**	–
LYM				
<1.6	**-**	Reference	**-**	–
1.6-4	0.620	1.19 (0.61–2.36)	**-**	–
≥4	0.996	0.00 (0.00-Inf)	**-**	–
HGB				
<120	**-**	Reference	**-**	Reference
120-160	**0.013**	0.45 (0.24–0.84)	0.116	0.55 (0.26–1.16)
≥160	0.506	0.68 (0.22–2.10)	0.75	0.81 (0.23–2.89)
PLT				
<100	–	Reference	–	–
100-300	0.611	0.60 (0.08–4.34)	–	–
≥300	0.622	0.59 (0.07–4.75)	–	–
FIB				
2-4	**-**	Reference	**-**	Reference
≥4	**0.011**	2.15 (1.19–3.89)	**0.034**	1.97 (1.05–3.68)
ALB				
<40	**-**	Reference	**-**	Reference
40–55	**0.048**	0.51 (0.27–1.00)	0.551	0.80 (0.37–1.69)
AH				
<10	–	Reference	–	
10-20	0.431	0.80 (0.45–1.40)	–	–
≥20	0.817	0.90 (0.36–2.22)	–	–
ADR				
<1	**-**	Reference	**-**	–
≥1	0.290	0.53 (0.17–1.71)	**-**	–
FH				
<10	–	Reference	–	–
10-20	0.557	1.18 (0.68–2.01)	–	–
≥20	0.345	0.56 (0.17–1.87)	–	–
FDR				
<1	–	Reference	–	–
≥1	0.682	0.87 (0.46–1.66)	–	–
ARCO				
II	**-**	Reference	**-**	Reference
IIIA	0.562	1.22 (0.627–2.36)	0.473	1.28 (0.65–2.50)
IIIB	**<0.001**	3.22 (1.73–5.98)	**0.002**	2.72 (1.44–5.17)
JIC				
A	–	Reference	–	Reference
B	0.535	1.98 (0.23–16.91)	0.915	1.12 (0.13–9.91)
C1	0.975	1.03 (0.13–8.41)	0.679	0.64 (0.08–5.33)
C2	0.258	3.14 (0.43–22.83)	0.570	1.80 (0.24–13.74)
Screw				
No	–	Reference	–	–
Yes	0.124	1.52 (0.89–2.61)	–	–
PRP				
No	–	Reference	–	–
Yes	0.641	1.33 (0.41–4.36)	–	–
Stem cell				
No	–	Reference	–	–
Yes	0.662	0.64 (0.089–4.66)	–	–
ASA				
I	–	Reference	–	–
II	0.839	1.06 (0.62–1.80)	–	–
III	0.997	0.00 (0.00-Inf)	–	–
Factor				
Glucocorticoid	–	Reference	–	–
Alcoholic	0.182	1.50 (0.83–2.72)	–	–
Traumatic	0.759	0.85 (0.29–2.47)	–	–
Idiopathic	0.995	1.00 (0.42–2.37)	–	–
Smoking				
No	**-**	Reference	**-**	Reference
Yes	**0.019**	1.94 (1.11–3.37)	**0.007**	2.32 (1.26–4.24)
Hypertension				
No	**-**	Reference	**-**	Reference
Yes	**<0.001**	6.71 (2.86–15.72)	**0.002**	4.43 (1.71–11.48)
Diabetes				
No	–	Reference	**-**	Reference
Yes	**0.07**	3.71 (0.90–15.32)	**0.009**	7.62 (1.71–34.05)

### Validation of the nomogram model

The model calibration curve, verified internally by bootstrap resampling 1000 times, is shown in Figure 4. It is apparent from the calibration diagram that the predicted probability at three time points had an excellent fit with the actual survival situation, indicating that the model has excellent consistency. Among them, the predicted curve of 5-year survival time had the best overlap with the actual curve. The C-index of the nomogram model for predicting failure after FSG was 0.734 (95%CI 0.66–0.80), suggesting that the model had an acceptable discriminative ability. Furthermore, the area under the curves (AUCs) of the nomogram predicted for 5, 8, and 10 years were 0.727, 0.735, and 0.735, respectively (Figure 5), manifesting that the prediction model has good accuracy. The above results revealed that the nomogram can favorably predict the prognosis of patients who underwent FSG surgery.

Figure 4. The calibration curves for assessing the consistency of predicting the probability of arthroplasty in patients with osteonecrosis of the femoral head after fibula allograft propping. Favorable consistencies between the predicted and the actual survival probabilities are presented, for time points of 5 (A), 8 (B), and 10 years (C).

**Figure:**
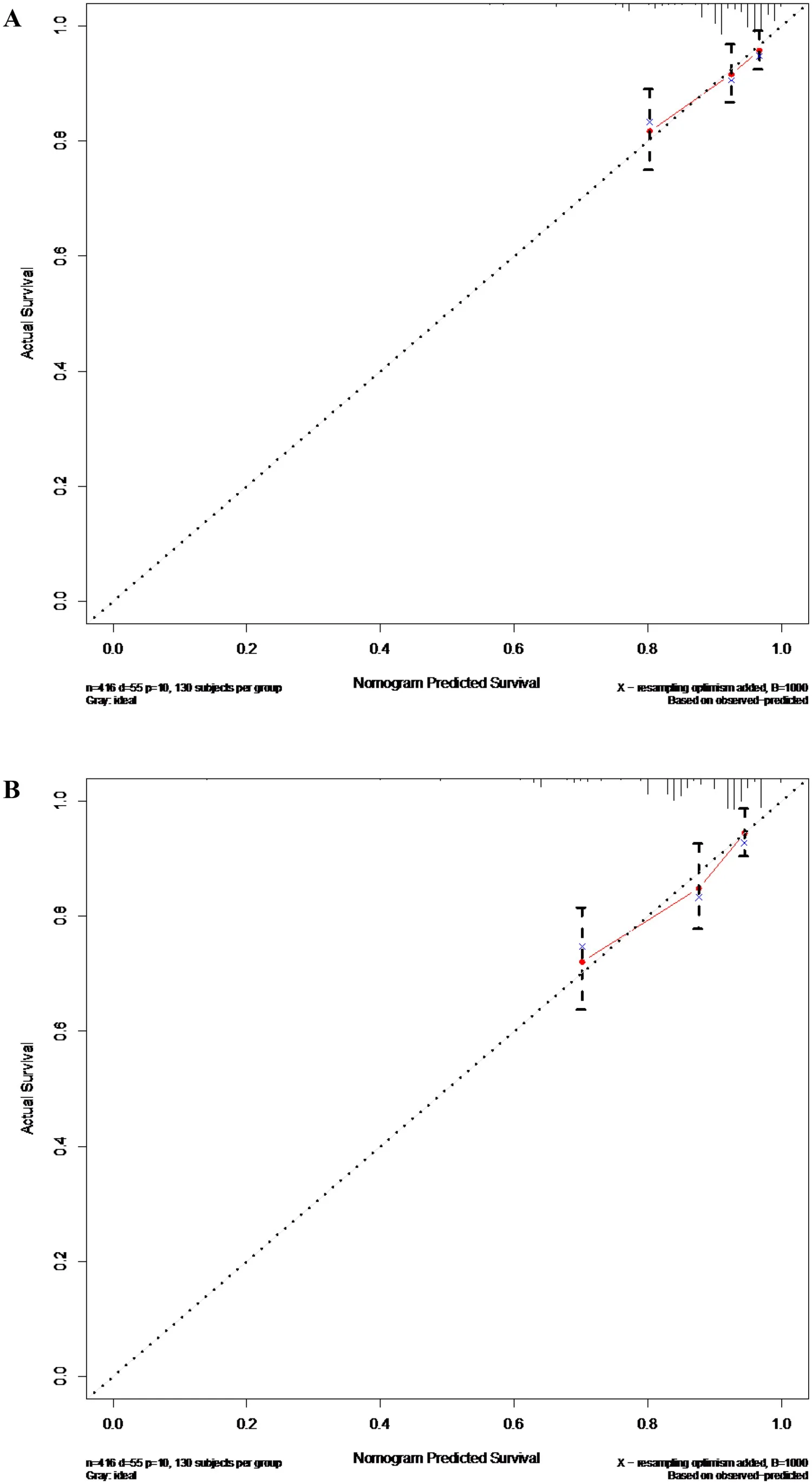

**Figure:**
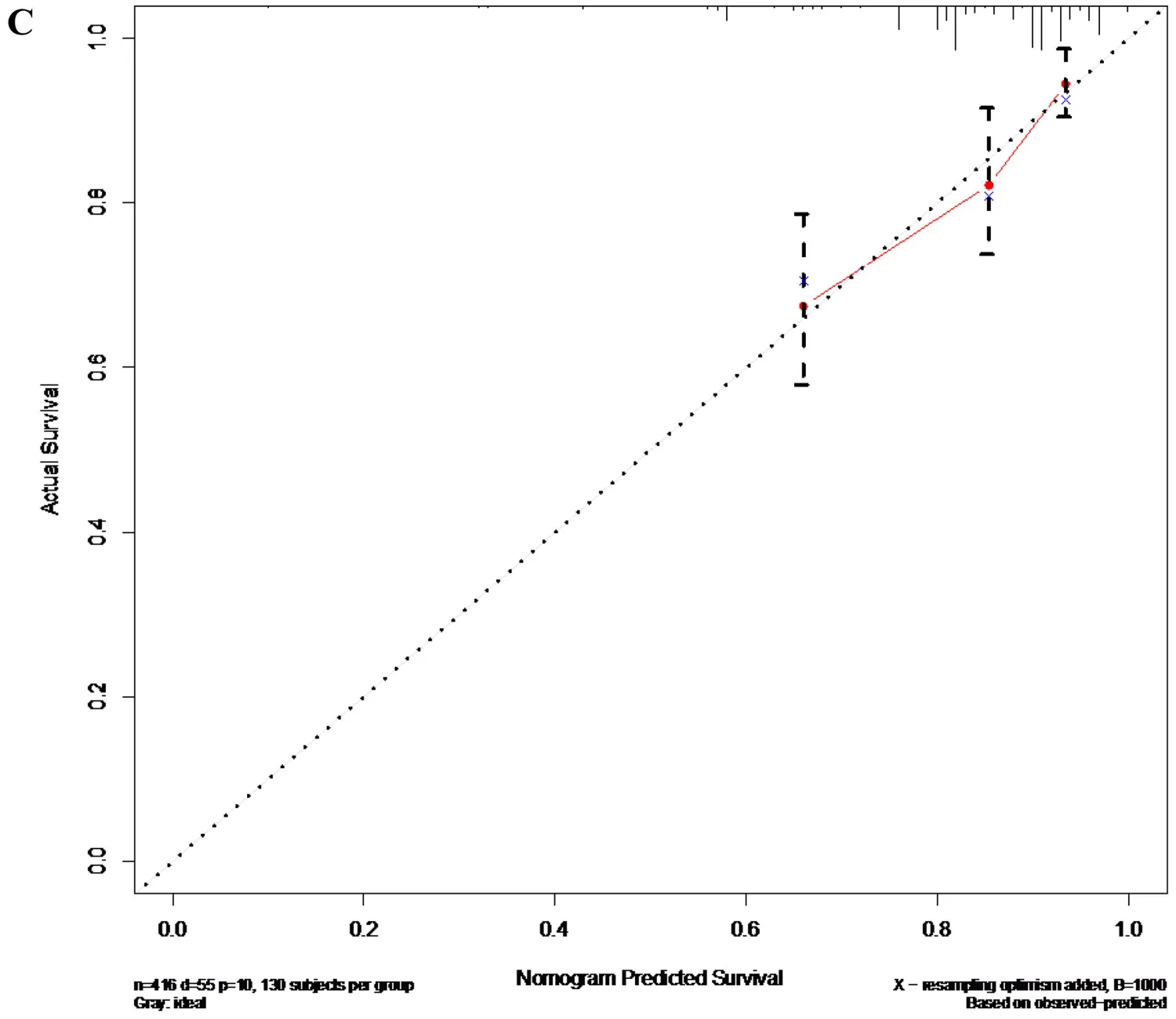

Figure 5. The ROC analysis of the predictive model for time points of 5 (A), 8 (B), and 10 years (C).

**Figure:**
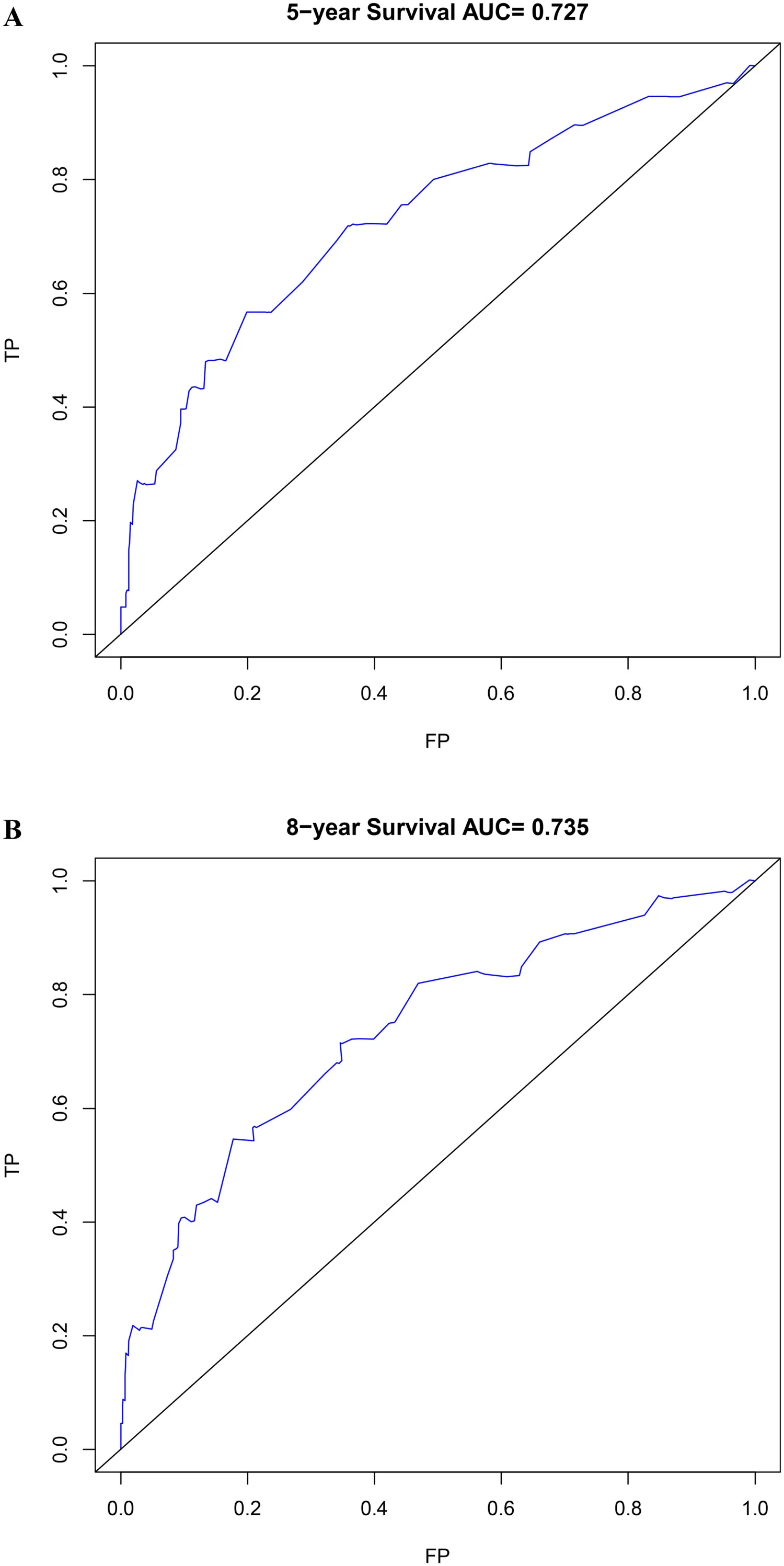

**Figure:**
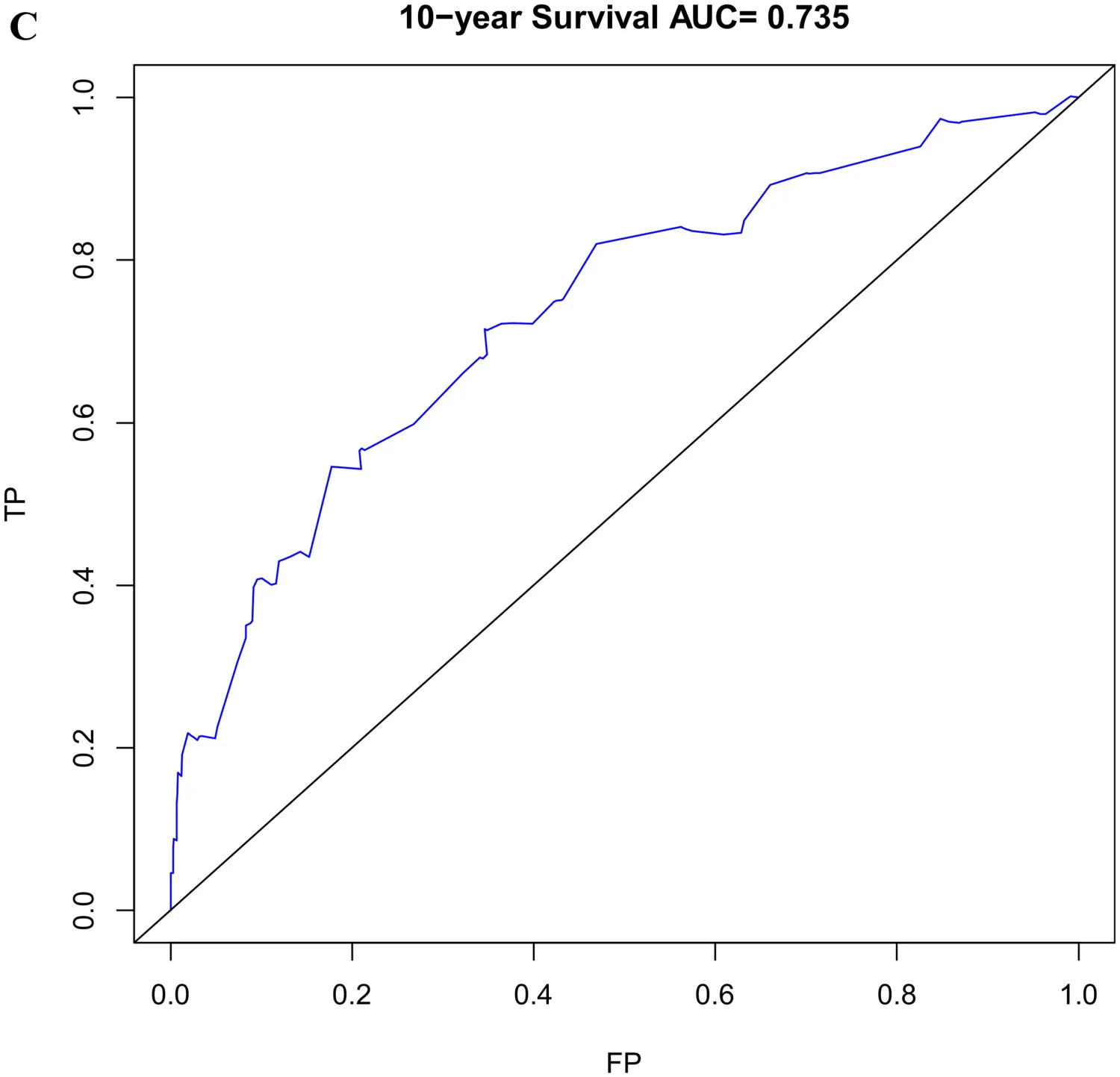

## Discussion

Experts recommend that hip joints be preserved as much as possible in active and young patients with ONFH [24]. Indeed, in the case of patients at different stages, there is still contention on which kind of hip-preserving surgery to select. At present, there are various methods of hip-preserving surgery with a high success rate. Whereas, there are more or fewer shortcomings. FSG is one of the non-vascularized bone grafting techniques, which has been improved from the Phemister technology proposed in 1949 [25]. To date, several studies have reported that the long-term survival rate of patients with ONFH who underwent FSG or even higher, but about 20% of patients were still facing further deterioration [18]. It is of great significance to accurately predict the outcome of patients after FSG to determine the optimal population for FSG treatment according to various factors. Hence, this study investigated the risk factors of failure and mid-long term survival rates in patients after FSG, and developed a dependable nomogram to predict the probability of THA after FSG in patients with ONFH based on age, smoking, FIB level, diabetes, ARCO stage, and JIC classification. To the best of our knowledge, this is the first study to develop a nomogram model to predict the survival rate after FSG in ONFH patients.

In terms of surgical procedures, FSG has become a more mature technology. Nevertheless, the success rate of surgery varies greatly in different institutions, and its indications have not been unified. It is a prevailing belief that the failure rate of hip-preserving surgery will increase with the extension of follow-up time. Sultan et al. had systematically reviewed 18 studies of non-vascular bone allografts from 2000 to 2018 [26]. Among them, 11 studies used the Phemister technique with the follow-up time ranging from 24 to 84 months, and the success rate of the operation ranged from 56 to 87%. In the study of 168 patients treated by core decompression combined with an allogeneic bone graft and followed up for 24 months, the overall survival rate was 81%, including 93.4% for stage IIB, 58.7% for stage IIC, and 86.5% for Stage IIIA [27]. In our study, the 5-year, 8-year, and 10-year survival rates of patients after FSG were 89.7%, 83.6%, and 82%, respectively. The results were generally similar to previous studies. When contextualizing our survival rates within the existing literature, instructive comparisons can be made. A systematic review reported a failure rate of 14.5% for head-preserving surgery in 394 hips with ONFH treated with fibular allograft over a mean follow-up of 49.06 months, a finding that is consistent with our 5-year survival rate [7]. In contrast, a long-term study from Taiwan with a 14-year follow-up reported an overall clinical success rate of only 65.5% for Phemister bone grafting in patients with non-traumatic ONFH, which is notably lower than our 10-year survival rate of 82% [28].

Our research identified that age, smoking, FIB, hypertension, diabetes, and ARCO stage are independent risk factors for failure after FSG. Subsequently, 7 predictors (including JIC classification) across three dimensions were incorporated into the nomogram model.

As for the analysis of risk factors, scholars generally agree that hip preservation is suitable for young ONFH, but the specific range of age is rarely discussed. Recent studies have reported that the optimal range of age for patients with impacted bone allograft is less than 40 years old [29]. This is similar to the results of our study. This is similar to our research results. Patients less than 40 years old have higher survival rates. Additionally, there is no direct study to investigate the relationship between smoking and failure after FSG. However, whether smoking causes or aggravates ONFH seems to be controversial. Previous basic studies had corroborated that nicotine in tobacco may cause arteriolar spasm and microcirculatory disorders in the femoral head [30]. An epidemiological study in Japan has conclusively shown that quitting smoking may help to effectively reduce the overall incidence of ONFH, indicating that there is a certain correlation between smoking and ONFH [31]. A randomized controlled study by Sakata documented that there was no significant correlation between smoking and ONFH [32]. A meta-analysis reported that the dose of smoking was positively correlated with the likelihood of developing ONFH [18]. Our results demonstrated that patients who smoke after FSG have a lower survival rate, which is consistent with that of Wen et al. There are a few studies on the relationship between serum markers and ONFH. The basic studies of Liu et al. had suggested that the precursor of fibrinogen α chain is a potential biomarker of hormonal ONFH [33]. Simultaneously, Cui et al. had noted that low LYM and hypertension were independent predictors of ONFH after internal fixation of Garden I femoral head fracture [34]. Similarly, patients with fibrinogen above normal (> 4) and hypertension are more likely to convert to THA after FSG in our series. Paradoxically, LYM is not an independent predictor in this research. The discrepancy was attributed to the difference in the normal range of LYM adopted in the present study [(1.1 − 3.2) × 10^9^/L] compared with that used by Cui et al. From a pathophysiological perspective, elevated fibrinogen reflects a hypercoagulable and pro-inflammatory state, which likely impairs microcirculatory reperfusion and graft incorporation within the ischemic femoral head. Concurrently, hypertension induces vascular endothelial dysfunction and disrupts blood flow autoregulation, creating an unfavorable environment for osteoblast activity and bone remodeling. These systemic perturbations may counteract the local mechanical stability afforded by FSG, thereby increasing the risk of non-union and progression to THA [19,35]. A study of a nomogram predictive model by Xu et al. confirmed that there was no significant correlation between diabetes and the incidence of ONFH after cannulated screws fixation [36]. This is the inverse of the results of our research. It should be noted that the *P* value of diabetes is only 0.07 in the univariate COX regression analysis of our study, but the significance is greatly increased after diabetes is included in the multivariate COX regression analysis. Certainly, this result also needs special basic research to verify.

Furthermore, Chen et al. determined that the less than 2 mm collapse of the femoral head and the intact lateral wall of the femoral head are indications of impaction bone allograft [29]. Wu et al. analyzed 29 patients who underwent the Phemister technique of two-strut allografts and found that ARCO stage and necrosis index ≥ 0.67 were independent risk factors for the outcome of THA. It is worth noting that the 2019 revised version of ARCO staging is used in our study, in which the difference between IIIA and IIIB is defined by the collapse of the femoral head > 2 mm [21]. Our results elucidated that patients with femoral head collapse > 2 mm are more likely to convert to THA after FSG. Surprisingly, there was no statistical difference for JIC classification, either in univariate or multivariate COX regression analysis. This is contrary to our clinical experience and previous research results. As a result, JIC classification was incorporated into the model to compensate for this limitation.

Additionally, from a biomechanical perspective, it was initially hypothesized that the use of lag screw fixation or the position of allogeneic fibular strips might affect the risk of failure after FSG. Unfortunately, there is no significant correlation between the use of lag screw fixation or the location of allogeneic fibular strips and the failure after FSG in this study, which may require further research. To bridge the gap between statistical prediction and clinical application, two representative cases were presented to demonstrate the practical utility of the nomogram in individualized risk assessment. Case 1: A 25-year-old male (ARCO II) with a predicted 5-year survival of 84.62%. Serial radiographs (Figure 6) showed preserved head sphericity and graft incorporation at 55 months follow-up. Case 2: Conversely, a 40-year-old male (ARCO III) with a predicted survival of <50%. Radiographs at 11 months, immediately prior to THA (Figure 7C), revealed progressive collapse and graft non-union, validating the model’s high-risk prediction.

**Figure 6.**
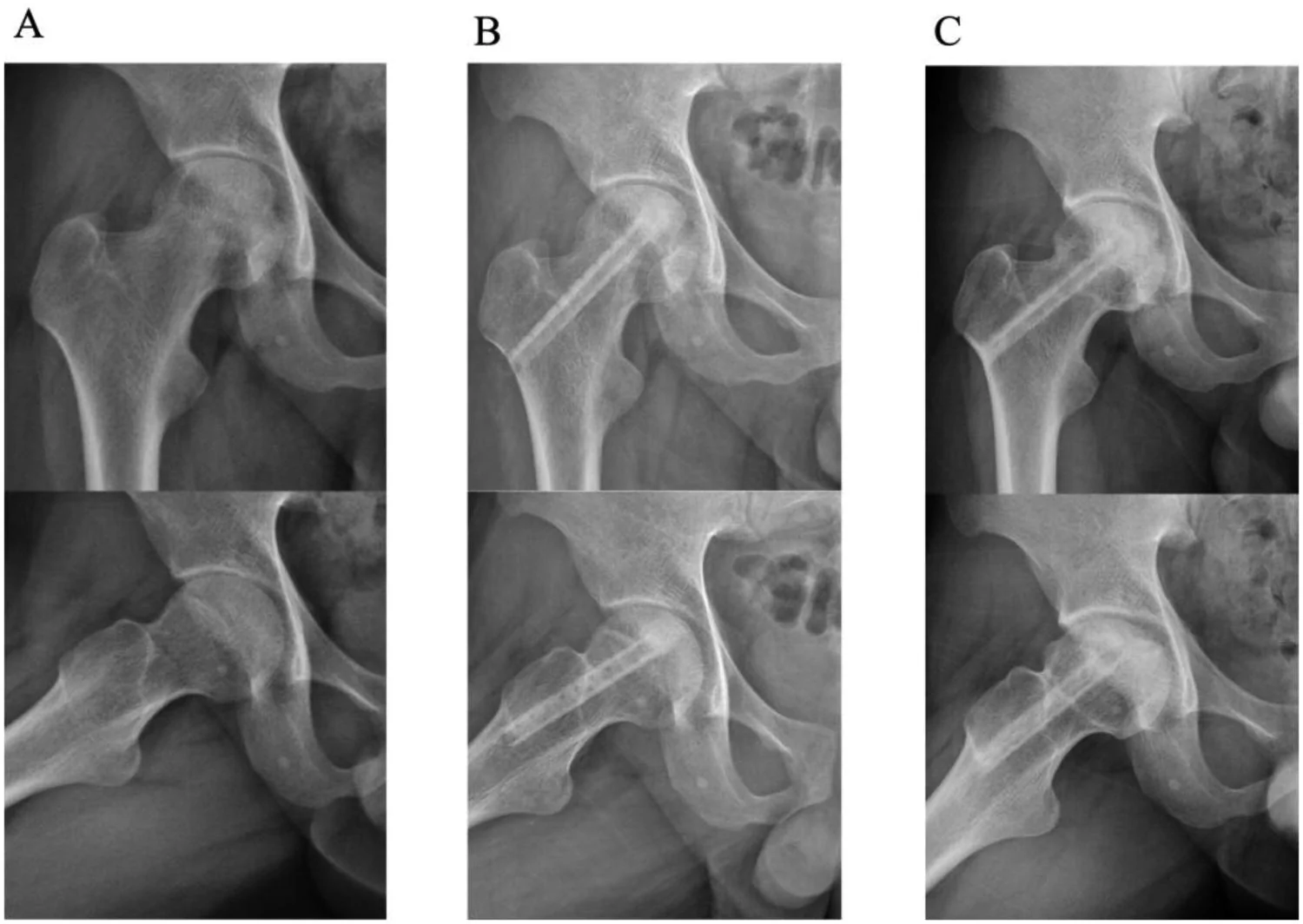
Serial radiographs of Case 1 (Successful FSG). A 25-year-old male with ARCO II ONFH. (A) Preoperative, (B) immediate postoperative, and (C) 55 months follow-up views showing preserved head sphericity and graft incorporation.

**Figure 7.**
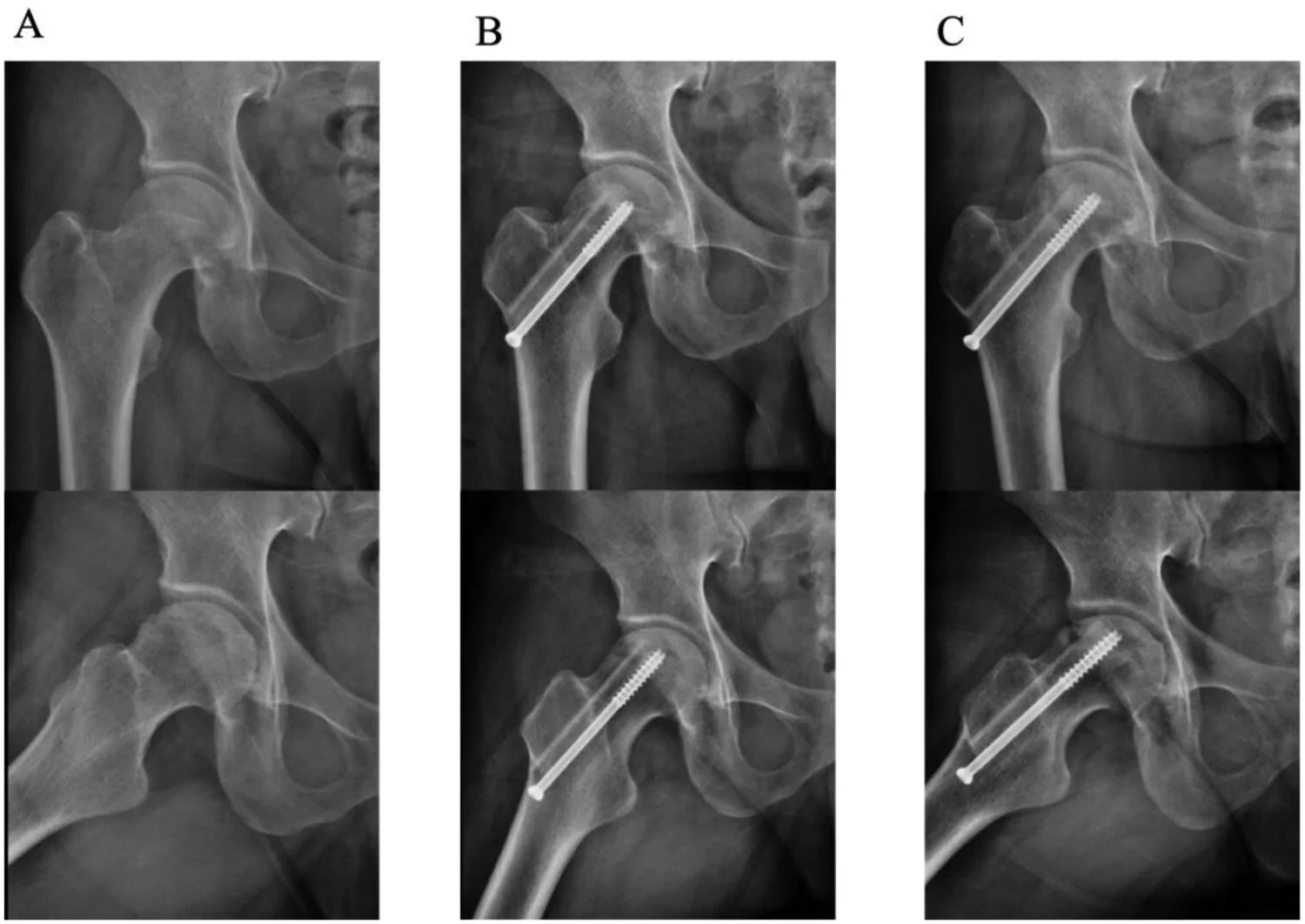
Serial radiographs of Case 2 (Failed FSG). A 40-year-old male with ARCO III ONFH. (A) Preoperative, (B) immediate postoperative, and (C) 11-month follow-up views (prior to THA) demonstrating progressive collapse and graft non-union.

Undoubtedly, there are also some limitations in this study. First and most importantly, the development and validation of our nomogram were conducted on a single-center, retrospective dataset. Although internal validation *via* bootstrapping yielded promising results, the model lacks external validation from independent patient cohorts at other institutions. Therefore, future multi-center studies with independent external validationare required to establish the generalizability of our model. Secondly, the time span of this retrospective study is nearly 10 years, which may have a slight impact on the surgical results with the gradual maturity of surgical techniques over time. Furthermore, although the core surgical protocol was standardized across the 10-year period, the involvement of multiple surgeons inherently introduces potential performance bias. Notably, the decision to use AO screws for fixation was based on individual surgeon preference rather than a rigid protocol. However, subgroup analysis revealed that the type of screw fixation did not significantly impact the primary outcome (THA conversion), mitigating concerns regarding this variability. Thirdly, some patients with short follow-up may be converted to THA in the future due as this study is based on COX regression. Hence, a longer follow-up is needed. Fourthly, specific non-traumatic subtypes (e.g. SLE, post-transplant) were not analyzed separately due to sample size constraints for each subgroup. Finally, the lack of data on corticosteroid dose/duration and ethanol use for the non-traumatic ONFH subgroup, a consequence of inconsistent documentation in our retrospective dataset. Future prospective studies are imperative to incorporate these critical risk factors.

## Conclusion

In summary, we identified that age, smoking, FIB, hypertension, diabetes, and ARCO stage were significantly related to the probability of THA after FSG. The nomogram prediction model was developed and verified using 7 prediction factors covering three dimensions. The internal verification shows that the model has acceptable discriminative ability and good accuracy. The nomogram model can play a valuable role in clinical application, effectively optimize perioperative management, and help doctors and patients avoid the risk of surgical failure.

## Supplementary Material

Supplementary Table1.docx

Supplementary Table2.doc

Supplementary Fig2.docx

Supplementary Figure 1.tif

## Data Availability

The data used to support the findings of this study are available from the corresponding author upon reasonable request.
