# Supplementary material for: Development and validation of a nomogram for predicting the probability of arthroplasty in patients with osteonecrosis of the femoral head after fibular strut grafting
Source: Ann Med. 2026 Oct 8;58(1):2734061. doi: 10.1080/07853890.2026.2734061 (PMC13656659; doi:10.1080/07853890.2026.2734061)
Supplement: Supplementary file 1 [file IANN_A_2734061_SM0112.docx]

477 patients with osteonecrosis of the femoral head (ONFH)

in The First Affiliated Hospital of Guangzhou University of Chinese Medicine who underwent fibular strut grafting (FSG)

from February 2009 to December 2018

61 patients were excluded:

Died: N = 5

Lost to follow-up: N = 48

Missing data: N = 6

Pathological fracture / other contraindications: N = 2

416 patients were included in the study analysis

**Supplementary Fig. 2.** Patient flow diagram. Patients with osteonecrosis of the femoral head who underwent fibular strut grafting between February 2009 and December 2018 were identified. After exclusions, 416 patients were included in the analysis. THA, total hip arthroplasty; ONFH, osteonecrosis of the femoral head; FSG, fibular strut grafting.
