# Supplementary material for: Development and validation of a nomogram for predicting the probability of arthroplasty in patients with osteonecrosis of the femoral head after fibular strut grafting
Source: Ann Med. 2026 Oct 8;58(1):2734061. doi: 10.1080/07853890.2026.2734061 (PMC13656659; doi:10.1080/07853890.2026.2734061)
Supplement: Supplementary file 2 [file IANN_A_2734061_SM0114.docx]

# Supplementary Table1 Univariate and Multivariate Analyses of Proportional Hazards Assumption

| **Variables** | **Univariate analysis P-value** | **Multivariate analysis P-value** |
| --- | --- | --- |
| Gender | 0.05 | - |
| Age | 0.87 | 0.84 |
| BMI | 0.39 | - |
| Side  WBC  LYM  HGB  PLT  FIB  ALB  AH  ADR  FH  FDR  ARCO  JIC  Screw  PRP  Stem cell  ASA  Factor  Smoking  Hypertension  Diabetes | 0.25  0.83  0.89  0.66  0.88  0.77  0.6  0.5  0.58  0.53  0.82  0.29  0.45  0.11  0.6  0.24  0.36  0.59  0.7  0.16  0.73 | -  -  -  0.49  -  0.86  0.28  -  -  -  -  0.14  0.55  -  -  -  -  -  0.67  0.16  0.56  P global=0.6 |
|  |  |  |
|  |  |  |
