# Supplementary material for: Development and validation of a nomogram for predicting the probability of arthroplasty in patients with osteonecrosis of the femoral head after fibular strut grafting
Source: Ann Med. 2026 Oct 8;58(1):2734061. doi: 10.1080/07853890.2026.2734061 (PMC13656659; doi:10.1080/07853890.2026.2734061)
Supplement: Supplementary file 3 [file IANN_A_2734061_SM0113.doc]

STROBE Statement—Checklist of items that should be included in reports of ***cohort studies***

|  | Item No | Recommendation | Reported on manuscript page / section |
| --- | --- | --- | --- |
| **Title and abstract** | 1 | (*a*) Indicate the study’s design with a commonly used term in the title or the abstract | **Title:** “Development and validation of a nomogram...”(retrospective cohort implied); **Abstract:** “retrospective study” |
|  |  | (*b*) Provide in the abstract an informative and balanced summary of what was done and what was found | **Abstract** (page 1–2): Background, Methods, Results, Conclusion |
| Introduction | | |  |
| Background/rationale | 2 | Explain the scientific background and rationale for the investigation being reported | **Introduction** (page 3–4): ONFH burden, FSG surgery, probability of arthroplasty after FSG |
| Objectives | 3 | State specific objectives, including any prespecified hypotheses | **Introduction** (page 5, lines 99–103): “determine risk factors... develop and validate a nomogram” |
| Methods | | |  |
| Study design | 4 | Present key elements of study design early in the paper | **Methods** (page 5, lines 106–107): “retrospectively collected data... retrospective observational cohort study” |
| Setting | 5 | Describe the setting, locations, and relevant dates, including periods of recruitment, exposure, follow-up, and data collection | **Methods** (page 5, lines 106–108): single institution; Feb 2009–Dec 2018; analyzed Mar 2025 |
| Participants | 6 | (*a*) Give the eligibility criteria, and the sources and methods of selection of participants. Describe methods of follow-up | **Methods** (page 5–6): inclusion/exclusion criteria; **Results** (page 11): 477 → 416; follow-up by telephone, outpatient, WeChat |
|  |  | (*b*) For matched studies, give matching criteria and number of exposed and unexposed | Not applicable (no matching) |
| Variables | 7 | Clearly define all outcomes, exposures, predictors, potential confounders, and effect modifiers. Give diagnostic criteria, if applicable | **Methods** (page 7–9): outcome = THA; predictors = age, smoking, FIB, hypertension, diabetes, ARCO, JIC; ARCO 2019 revised criteria, JIC classification |
| Data sources/ measurement | 8* | For each variable of interest, give sources of data and details of methods of assessment (measurement). Describe comparability of assessment methods if there is more than one group | **Methods** (page 7–9): EMR for baseline/labs; radiographs for ARCO/JIC; two blinded senior surgeons; third adjudicator for discrepancies |
| Bias | 9 | Describe any efforts to address potential sources of bias | **Methods** (page 8–9): standardized surgical protocol, blinded radiology assessment, imputation for missing data; **Discussion** (page 19): limitations acknowledged |
| Study size | 10 | Explain how the study size was arrived at | **Methods/Results** (page 5, 11): consecutive eligible patients from Feb 2009–Dec 2018; final n=416 |
| Quantitative variables | 11 | Explain how quantitative variables were handled in the analyses. If applicable, describe which groupings were chosen and why | **Methods** (page 9–10): age grouped (40–55y, >55y); FIB ≥4 g/L; ARCO stage; JIC classification; left-closed right-open principle |
| Statistical methods | 12 | (*a*) Describe all statistical methods, including those used to control for confounding | **Methods** (page 9–10): univariate Cox (P<0.1), multivariate Cox (P<0.05), Kaplan-Meier, nomogram, C-index, ROC, calibration, bootstrap 1000 |
|  |  | (*b*) Describe any methods used to examine subgroups and interactions | **Discussion** (page 18): subgroup analysis by screw fixation type; ARCO/JIC subgroups |
|  |  | (*c*) Explain how missing data were addressed | **Methods** (page 9–10): mean imputation for continuous variables; mode imputation for categorical variables |
|  |  | (*d*) If applicable, explain how loss to follow-up was addressed | **Methods/Results** (page 5, 11): 48 patients lost to follow-up were excluded; reported in flow |
|  |  | (*e*) Describe any sensitivity analyses | Internal validation via 1000 bootstrap resampling was performed to assess the stability of the model (Methods, page 10; Results, page 12). |
| Results | | |  |
| Participants | 13* | (a) Report numbers of individuals at each stage of study—eg numbers potentially eligible, examined for eligibility, confirmed eligible, included in the study, completing follow-up, and analysed | **Results** (page 11, lines 222–226): 477 identified → 416 included → 55 THA; **Figure 8** (flow diagram) |
|  |  | (b) Give reasons for non-participation at each stage | **Results** (page 11, lines 230–234): death (5), lost to follow-up (48), missing data (6), pathological fracture (2) |
|  |  | (c) Consider use of a flow diagram | **Figure 8** (to be added) |
| Descriptive data | 14* | (a) Give characteristics of study participants (eg demographic, clinical, social) and information on exposures and potential confounders | **Results** (page 11–12); **Table 1**: age, sex, BMI, smoking, hypertension, diabetes, ARCO, JIC, lab values |
|  |  | (b) Indicate number of participants with missing data for each variable of interest | **Methods** (page 9–10): imputation described |
|  |  | (c) Summarise follow-up time (eg, average and total amount) | **Results** (page 11): mean follow-up 76.8 ± 37.2 months |
| Outcome data | 15* | Report numbers of outcome events or summary measures over time | **Results** (page 11–12): 55 THA; 5-year 89.7%, 8-year 83.6%, 10-year 82% |
| Main results | 16 | (*a*) Give unadjusted estimates and, if applicable, confounder-adjusted estimates and their precision (eg, 95% confidence interval). Make clear which confounders were adjusted for and why they were included | **Results** (page 12); **Table 2**: univariate and multivariate Cox HR with 95% CI; confounders: age, smoking, FIB, hypertension, diabetes, ARCO |
|  |  | (*b*) Report category boundaries when continuous variables were categorized | **Methods** (page 9–10); **Table 2**: age (40–55y, >55y), FIB (≥4 g/L) |
|  |  | (*c*) If relevant, consider translating estimates of relative risk into absolute risk for a meaningful time period | **Results** (page 12); **Figure 2** (nomogram): absolute survival probabilities at 5, 8, 10 years |
| Other analyses | 17 | Report other analyses done—eg analyses of subgroups and interactions, and sensitivity analyses | **Results** (page 12–13): C-index (0.734), AUC (0.727–0.735), calibration curves; **Discussion** (page 18): screw fixation subgroup |
| Discussion | | |  |
| Key results | 18 | Summarise key results with reference to study objectives | **Discussion** (page 13, first paragraph): “this study investigated risk factors... developed a dependable nomogram” |
| Limitations | 19 | Discuss limitations of the study, taking into account sources of potential bias or imprecision. Discuss both direction and magnitude of any potential bias | **Discussion** (page 19–20): single-center, retrospective, no external validation, 10-year span, multiple surgeons, short follow-up, missing corticosteroid/ethanol data |
| Interpretation | 20 | Give a cautious overall interpretation of results considering objectives, limitations, multiplicity of analyses, results from similar studies, and other relevant evidence | **Discussion** (page 13–19): compared with literature; biological plausibility for each predictor |
| Generalisability | 21 | Discuss the generalisability (external validity) of the study results | **Discussion** (page 19): “future multi-center studies with independent external validation are required” |
| Other information | | |  |
| Funding | 22 | Give the source of funding and the role of the funders for the present study and, if applicable, for the original study on which the present article is based | **Fundings** (page 20–21): National Natural Science Foundation of China (82374484), etc. |

*Give information separately for exposed and unexposed groups.

**Note:** An Explanation and Elaboration article discusses each checklist item and gives methodological background and published examples of transparent reporting. The STROBE checklist is best used in conjunction with this article (freely available on the Web sites of PLoS Medicine at http://www.plosmedicine.org/, Annals of Internal Medicine at http://www.annals.org/, and Epidemiology at http://www.epidem.com/). Information on the STROBE Initiative is available at http://www.strobe-statement.org.
